# The experiences of ‘Suicide Prevention Champions’ in the UK National Health Service (NHS): A qualitative investigation

**DOI:** 10.1371/journal.pmen.0000623

**Published:** 2026-08-10

**Authors:** Abbie McDonogh, Pui Lok Joshua Yiu, Harpreet Gill, Jo Billings

**Affiliations:** 1 Division of Psychiatry, University College London, London, United Kingdom; 2 North London NHS Trust, London, United Kingdom; Manchester Metropolitan University, UNITED KINGDOM OF GREAT BRITAIN AND NORTHERN IRELAND

## Abstract

Suicide is a leading cause of preventable death globally with 17 people dying by suicide every day in the United Kingdom (UK). A growing body of research and governmental policies has been devoted to developing and implementing suicide prevention interventions, including training healthcare staff about suicide. However, more research is needed around staff experiences of the training and delivery of these interventions. We aimed to address this gap in the literature in the context of a voluntary ‘Suicide Prevention Champion’ role trialled in two National Health Service (NHS) Trusts in the UK. The role involves existing inpatient mental healthcare staff receiving specific Applied Suicide Intervention Skills Training and access to a Community of Practice composed of monthly meetings and email support. Semi-structured interviews were conducted with a purposive sample of 10 Champions. Reflexive Thematic Analysis was used to analyse the transcripts of the interviews. We identified seven inductive themes, organised into three deductive domains; ‘Driving Factors and Barriers’, ‘Impacts of Training’ and ‘Experience of the Role’. The Champions generally spoke highly of the role and training. However, important developments were noted; including a desire for greater clarity around the role and a suggestion that the Champions could inform a more specific NHS training. These findings are hoped to contribute to the ongoing development of the Champion role within the NHS.

## Introduction

Suicide is a tragic and preventable loss of life and a leading cause of death globally, with profound effects on individuals, families, and communities [1,2]. Every day 17 people in the UK die by suicide [3]. Although, this figure likely underestimates the true impact of suicide in our society as it neglects to consider the number of suicidal attempts which did not result in death. According to the 2025 Adult Psychiatric Morbidity Survey rates of reported attempted suicides are increasing [4]. In 2000, 0.5% of those aged 16–74 surveyed in England reported having attempted suicide in comparison to 1% in 2023/24. These figures suggest that the true impact of suicide in the UK may extend beyond our current predictions.

The term suicide specifically refers to self-inflicted, intentional, and fatal deaths [5]. Yet, it can be helpful to conceive of suicide as a continuum including attempts, ideation, and behaviours [6]. Understanding how and why people progress along this continuum and intervening accordingly is considered vital for suicide prevention efforts [6]. Equally, while completed suicide rates in the UK are highest amongst males, those aged 50–54 years of age, and people living in the most deprived areas, it is important to remember any demographic can be at risk of suicide and this risk can change rapidly [3].

Suicide prevention has been an ongoing focus for the UK government over the past decade. In 2016, the NHS Five Year Forward View for Mental Health committed to reducing suicide rates in England by 10% [7]. However, by the time the plan ended there had been no statistically significant difference in suicide rates, and in fact, rates have risen since [8]. This fact highlights the importance of ensuring that the suicide prevention strategies we employ are effective. More recently, in 2019, the government allocated £57 million to suicide prevention. Also, in 2023, the government’s 5-year cross sector strategy for suicide prevention in England was published and discussed specific strategies, including restricting the methods used for suicide (also known as ‘means restriction’) and staff training [8].

### Suicide prevention strategies

Suicide prevention strategies can be direct or indirect. Direct interventions, such as means restriction, have a clear role in prevention efforts [9]. The use of means restriction was recommended by the National Institute for Health and Care Excellence (NICE) to address railway suicides, which were thought to account for 293 fatalities in Britian between April 2024 and March 2025 [9,10].

Indirect strategies, such as staff or ‘gatekeeper’ training, are also vital components of suicide prevention efforts. The term ‘gatekeeper’ is used in suicide prevention literature to refer to people who may have contact with those at risk of suicide, such as healthcare workers. [11]. A study of 1721 people who died by suicide in Wales between 2001–2017, found that 31.4% had contacted health services in the week before their death [12]. Similarly, of the 17 people who die from suicide in the UK every day, five are estimated to have been in contact with mental health services [3]. Thus, in the UK, mental health staff and health professionals could be viewed as critical ‘gatekeepers’ with key opportunities for intervention. The results of two recent systematic reviews (synthesising the results of 23 articles and 97 randomised controlled trials, respectively) found training healthcare professional ‘gatekeepers’ improved their knowledge and increased their efficacy in suicide prevention [13,14].

### Assessing and formulating suicidal risk in the UK’s National Health Service

In the UK, the NHS is organised in a structured way, and suicidal risk assessments take place across all levels of the system; including in primary, secondary, and specialist care [15]. The National Institute for Care and Excellence is a government body that provides evidence-based guidelines to improve health and social care. NICE guidance is available to guide suicide risk assessments at each level of the UK’s health system and beyond, including in academic and legal contexts [15].

The expected depth of the assessment of suicide risk differs at each level. Non-healthcare staff are encouraged to observe signs and refer to services if needed while in primary care, general practitioners (GPs) and community pharmacy staff are told to use consultations to ask about thoughts of suicide, self-harm, and access to potentially life-threatening substances. Mental health staff are required to conduct full psychosocial assessments including the person’s current intent, psychiatric history and strengths to develop a safety plan and formulate the person’s risk of suicide and self-harm [15].

This shift towards personalised risk formulations replaces past reliance on risk ratings, in which stratified scales were used to evaluate suicidal risk [16]. NICE guidance currently advises against the use of such scales as they have been evidenced to have limited predictive validity and thus are inappropriate to address the dynamic nature of suicidal risk [15]. Of those who died by suicide and were in contact with healthcare professionals in the year before their death, 80% were rated as ‘low’ or ‘no’ risk [3]. The updated emphasis on professional skills such as information gathering, relationship building and clinical judgement to assess suicidal risk highlights the importance of having trained staff who feel confident in working with suicide in place at all levels of the NHS [16].

### Staff experiences of suicide prevention training and assessing suicide

There is a lack of research into staff experiences of training in and delivering suicide prevention interventions. Research has suggested that some healthcare professionals fear talking about suicidal ideation with patients in case they make it worse [17,18]. Sufficient training in suicide prevention has been shown to significantly increase staff confidence in working with suicidal patients [19]. Yet, what constitutes sufficient training is unclear and training programs vary greatly in length, style, and measured outcomes [19]. A recent UK qualitative study of forty-one people indicated that some participants viewed General Practitioners training in and knowledge of suicide as limited and this made them less likely to tell their GP if they were thinking about suicide. One participant commented ‘I felt my medical needs were met, but not necessarily my mental health needs’ while another reflected ‘I wish [the GP] would just say suicide’ [20, p.7]. This research indicates that any suicide prevention training provided to healthcare professionals may be insufficient [19]. Thus, it appears essential to investigate staff experiences of suicide prevention trainings to assess the efficacy of the gatekeeper interventions which are being delivered. It is also important to recognise that healthcare staff can be as vulnerable to the emotional nature of suicide as the general population. Patient suicides have been associated with mental health difficulties, loss of confidence, and career change contemplations among NHS staff [18,21]. Equally, staff may encounter suicidal behaviours within their team, families, and friends, as well as from patients. In the first half of 2024, the number of nurses seeking support for suicidal ideation was 76% higher than for the same period in 2023, with workplace pressure named as a contributing factor [22]. To support staff wellbeing and work towards the NHS priority of suicide prevention, it is important to assess the experiences of staff in the training and delivery of these interventions [6].

### The ‘Suicide Prevention Champion’ role

In 2022, a voluntary ‘Suicide Prevention Champion’ role was established in two London NHS mental health Trusts. The role was open to existing inpatient mental healthcare staff of multi-disciplinary backgrounds, to undertake alongside their existing work, and consisted of two components; training and monthly meetings.

Firstly, the Champions were required to attend the Applied Suicide Intervention Skills Training (ASIST), a group-based training which took place over two days. ASIST is a generic training aimed at increasing participants’ comfort in talking about suicide, their knowledge of sensitive terms, and their ability to recognise warning signs of suicidal ideation and create safety plans in response. ASIST has taught and experiential components, including role-plays and since its creation in 1983, the training has been regularly reviewed and attended by over two million people [23]. In America, the ASIST program was used as a gatekeeper intervention across 21 states. A naturalistic pre-post test design was conducted to assess the impact of the program and found significant changes in all beliefs about suicidality including self-efficacy and perceived controllability among their 434 participants [24]. Similarly, of 401 multi-agency professionals in Glasgow who received the ASIST program through the Glasgow City Suicide Prevention Partnership, 100% said they would recommend the training [25].

Secondly, the Champions were required to attend monthly ‘Community of Practice’ (CoP) meetings. The ASIST programme was created to work best when delivered as part of a community-based approach [26]. Thus, the CoP was established as a space where the Champions could share new risk management resources, engage in roleplays, evaluate new interventions, and have group discussions, where needed, given the potential emotional impact of the role. The CoP meetings were scheduled to take place at alternating days, times, and modes of delivery (in-person or online) and people with lived experience were also invited to the meetings to share their input on best practice guidelines and changes to policy and legislation. The Champion role was headed by three senior members of staff known as the ‘leads of the COP’. The ‘leads of the COP’ were responsible for structuring these monthly meetings, organising when they and the ASIST training would take place, and recruiting for the role. It was hoped that the combination of ASIST and the CoP within this Champion role would be a positive step towards supporting healthcare professionals and reducing the rates of suicide in the NHS.

### Aims and objectives

The present study aimed to investigate the experiences of staff volunteering as “Suicide Prevention Champions” in the two London NHS mental health Trusts. In doing so, we hoped to address the lack of research on staff experiences of suicide prevention interventions. Evaluating the role’s development, delivery, and training offered the opportunity to identify areas for improvement, both for the effectiveness of the role and staff wellbeing. Further, enabling staff to co-produce this new role through the recommendations of this study is seen as vital for the utilisation of this, and similar staff training interventions, in the future.

## Materials & methods

### Design

The study employed an exploratory qualitative design using semi-structured interviews to investigate the experiences of inpatient mental health professionals working as ‘Suicide Prevention Champions’.

### Ethics

Ethical approval was granted by the research ethics committee at University College London (24813/001). The study was registered as a service evaluation with the two inner London NHS mental health Trusts in which it was set. All potential participants were provided with a Participant Information Sheet and gave informed consent in advance of the interview. Interview transcripts were pseudoanonymised and to reduce the risk of deidentification, participants will be referred to by those pseudonyms within this paper. Potentially identifiable information, including participant’s age, gender, and job title, will only be presented in an aggregated format.

### Participants and procedures

All staff trained as ‘Suicide Prevention Champions’ in two inner London NHS Trusts were informed about the study by email, inviting those interested to email the research team. Up to two follow-up emails were sent to enhance recruitment without being coercive.

Interviews were conducted at a mutually convenient time online via Microsoft Teams. Interviews were conducted in a semi-structured format to focus on core elements of the participants’ experiences while allowing for further exploration of interesting points of discussion [27]. The interview guide was developed by the research team with the support of the leads of the CoP. The leads of the CoP were not eligible to participate in this study due to their responsibilities in establishing and managing the role, which was thought to potentially influence their responses. The interview guide aimed to explore the driving factors and expectations that led participants to take on this voluntary role, experiences of the training, and challenges and satisfactions within the role (See S1 File).

All interviews were recorded and transcribed within a week of taking place by a professional transcription service in line with GDPR regulations. Participants were invited to check the completed transcript of their interview.

### Analysis

Reflexive thematic analysis was chosen for the analysis to enhance transparency as this methodology encourages researchers to consider their impact on the data [28]. The first author, A.M., kept a notebook to record her feelings and assumptions after each interview and following any major methodological decisions with the project team. This process of self-interrogation aided awareness of A.M.’s presence within the study. A.M. sought to identify inductive themes by immersing herself in the data, conducting, transcribing and pseudonymising the interviews, and noting any concepts which could be interesting and relevant to the research questions. A.M then created organising domains deductively, based on their knowledge of the literature, the research questions, and the interview guide.

Once each transcript was approved by those participants who consented to receive it, it was uploaded to NVivo 14 and all sections of the transcripts with information relevant to the research question were coded. The list of codes was refined by comparing new codes to existing ones and re-reading the transcripts repeatedly to consider whether any could be expanded, collapsed or renamed. A.M., then used a whiteboard, multicoloured pens, and Post-its to visualise how these codes may work together and to create themes. J.B.compared one transcript to the final list of themes developed by A.M. to assess coherency.

Additionally, to encourage iterative theme development, another member of the research team, J.Y, was asked to review two transcripts and actively create a list of themes and codes. The aim of consulting J.Y. was not to reach a consensus but to acknowledge that the rest of the analysis team, A.M., J.B., and H.G. are women from the UK and Ireland and these social locations may have impacted their interpretation of the data. As a male from Hong Kong, we hoped J.Y. could offer a fresh perspective, provoke discussion, and interrogate the confirmability and credibility of the conclusions drawn [29].

### Reflexivity

It is vital to consider our social locations and pre-existing ideas, as these experiences of the research team cannot be disentangled from the data [29]. In the pursuit of transparency, J.B. and H.G. are Consultant Clinical Psychologists working within the NHS and public sector, while A.M. and J.Y. were Master’s students in Clinical Mental Health Sciences at the time of data collection. J.B. and H.G.’s professional experience as NHS clinicians may have influenced how the research topic was approached and interpreted. In contrast, A.M. primarily approached the research from the perspective of a healthcare user rather than provider, although both A.M. and J.Y. had previous volunteering experience in mental health organisations, including services supporting individuals experiencing suicidality. These experiences, which occurred outside the NHS context, may have shaped their assumptions about the Champion’s role and training prior to data collection.

One member of the research team (H.G.) was employed in one of the NHS Trusts where the study took place, meaning some participants may have been their colleagues. To manage this potential conflict of interest, H.G.’s role was limited to facilitating initial contact with Communities of Practice leads for the purposes of recruitment. She had no access to information about who expressed interest in participating and was not involved in data analysis.

Several members of the research team have personal experiences of suicide within their families, and all were motivated to engage in this research due to a shared interest in the wellbeing of staff supporting individuals experiencing suicidality. Throughout the research process, reflexive practices, including reflective journaling, were used by J.B., A.M., and J.Y., to identify and critically consider preconceptions and emotional responses arising during interviews and analysis. For example, A.M. reflected that she did not initially expect the breadth of challenges the Champions described in balancing the voluntary role with their professional and personal responsibilities. A.M. considered that unlike the Champions, her prior voluntary experience had never taken place at her place of employment. In possibly overidentifying with the Champions, A.M. noted that she may have been overlooking important elements of their experience. Recognising these perspectives helped the team remain attentive to how their positionality and interpretations were embedded within the research process.

## Results

### Demographics

Eleven staff members from the NHS Trusts volunteered to participate in this study. One individual withdrew due to illness. Of the remaining 10 participants, eight identified as female and eight as ethnically White. Further information about participants’ ethnicities and job roles is displayed in Table 1. Participants’ ages ranged from 30 to 61 years (*M* = 57), and self-reported years of clinical experience ranged from 0 to 26 (*M* = 12.4).

**Table 1:** Participants’ ethnicities and job roles.

Demographic			*n*
Ethnicity	White	British	5
		European	2
		Australian	1
	Black	African	1
	Asian	Indian	1
Job role	Peer Coach		1
	Clinical/Counselling Psychologist		2
	Allied Health Professional (AHP)		2
	Health Care Professional (HCP)		5

Interviews took place between July 31st and August 22nd, 2023, and lasted from 24 to 52 minutes (*M* = 37.1). Participants were at various stages of the Champion role when interviews were completed. One participant had yet to attend the ASIST programme, while another had completed the training but had not yet attended any CoP meetings. Nevertheless, all interviewees were included in the analysis to capture the full breadth of experiences at each stage of the role and training.

### Reflexive thematic analysis

Seven inductive themes were identified through reflexive thematic analysis, each representing a core element of the Champions’ experience (Terry et al., 2017). These themes include the passion which motivated them towards the role and the professional barriers which inhibited them, the organisational challenges they faced, and the community of support they received. The themes also encompassed implications for participants’ confidence in and views of discussing suicide, and their hoped developments for the role going forward.

Eight subthemes were also identified inductively, as shown in Table 2. A sub-theme details a unique element of a theme, while sharing the same central organising concept. Three domains were created to organise the themes and sub-themes.

**Table 2:** Deductive domains and inductive themes and sub-themes.

Domain	Theme	Subtheme
Driving Factors and Barriers	Passion	Personal Experience
		Personal Interest
	Professional Barriers	
Impacts of Training	Self-Efficacy	
	Value of Discussion	Fears about Discussing Suicide
		Learning from NHS-specific Examples
Experience of the role	Community of Support	
	Organisational Issues	Time Commitment and Scheduling Clashes
		Desire for Clarity and Structure
	Developments	Promotion within Teams
		Dynamic Approach

### Driving factors and barriers

The domain ‘Driving factors and barriers’ captures both the motivations that led participants to engage with the Suicide Prevention Champion role and the structural challenges influencing access to the role and training. Two themes were identified within this domain: ‘Passion’ and ‘Professional barriers’.

#### Passion: Personal experience.

Personal experiences of suicidality drove many Champions to partake in the role and training. Several participants had lost loved ones to suicide, while others had experienced suicidal ideation themselves. The emotional impacts of these experiences varied across interviews. For instance, Holly, a HCP, felt *“triggered”* when she noticed signs of suicidality in service users who resembled her lost loved one. Alternatively, others believed their experiences had desensitised them, removing the shock factor around suicide and enhancing their resilience and ability to work as Champions. Anton, a HCP, acknowledged his experience had motivated him to become a Champion but recognised that it could affect people differently

*“Some people could instead say, ‘I don’t wanna hear any suicide (...), I don’t wanna talk about it’. I didn’t take it that way. For me, it was like, no, I don’t want this - what happened to my [redacted] - I don’t want it to happen to other people”*

All participants with personal experiences of suicide reported an enhanced sense of connection and empathy when interacting with the families of service users who had died by suicide. Chloe, a HCP, stated, *“I believe [the emotions] are processed. But in some way, I believe there is something familiar. Something that involves me and I can’t ignore”*.

Personal experiences of suicidality appeared to strongly motivate engagement with the Champion role while shaping participants’ empathy and emotional connection to suicide prevention work.

#### Passion: Personal interest.

A number of Champions described a pre-existing interest in suicide prevention, evident from their involvement in related research and voluntary roles. This personal interest drove many interviewees to self-nominate for the role after seeing generic email advertisements sent throughout the Trusts. Additionally, one participant was nominated by their manager due to their interest in suicide prevention. Kristin, a HCP, explained how her interest influenced her engagement with the work

*“I’m very passionate about suicide, in particular, in terms of the mental health field. So, that naturally helps me in terms of just being present about it, understanding how it might affect me at any point, what do I need, how do I respond to those types of situations.”*

These accounts suggest that for several participants, their pre-existing passion and interest in suicide prevention played an important role in motivating their participation as Champions.

#### Professional barriers.

Barriers to accessing the Champion role and training seemed to vary based on participants’ job titles and which area of the Trusts they worked in. Some non-managerial or junior staff and those in alternative clinical roles, including Mark, a Peer Coach, discussed barriers in the recruitment process

*“I emailed them straight away and said, ‘Right. I wanna be involved’. They said, ‘No you’re not a clinician’. I said, ‘Well, that’s ridiculous’, and kind of explained my sort of viewpoint to them. It then had to go for consideration and had to be supported by my line manager, (...) so it took a lot of fighting.”*

Other participants described structural barriers to accessing the training. For instance, Amaya, a HCP, could only access the ASIST programme if they adopted the Champion role. Amaya feared this requirement could prevent staff with limited time from learning about suicide prevention. However, experiences varied across teams as Holly, also a HCP, noted that while she was the only Champion on her team, many of her coworkers had completed the ASIST programme.

These findings suggest that organisational structures and eligibility criteria can be inconsistent across teams and could limit access to both suicide prevention training and the Champion role.

#### Impacts of training.

The domain ‘Impacts of training’ captures participants’ experiences of the ASIST programme and its influence on their confidence and approach to discussing suicide.

#### Self-efficacy.

Completing the ASIST programme generally enhanced the self-efficacy of even experienced participants. As Amaya said, *“A lot of my role is doing [Suicide Prevention Champion work] anyway. Because I’m dealing with a lot of risk day-to-day, but [the ASIST programme] helped me to feel better prepared.”*.

Losing a patient to suicide or visiting the coroner’s court as a team were identified as factors which could knock a person’s self-belief. Additionally, participants with many years of experience worried new guidance on suicide prevention had emerged since they had been trained. Thus, the training seemed to reassure staff of their suicide prevention knowledge. Sylvia, a AHP, stated, *“I’m coming at it from a perspective now of, like, ‘Actually, I have done this training. I do feel more confident in, yes, what I can offer people.’”.*

The ASIST programme appeared to strengthen participants’ confidence while validating their existing approaches to suicide prevention.

#### Value of discussion: Fears about discussing suicide.

Although many of the Champions regularly worked with suicidality, several participants admitted they had been scared to discuss suicide before the ASIST programme. Holly believed that as the Champions were human, conversations about suicide would always carry an element of fear. Alternatively, Sam, an AHP, believed his fears were related to common myths about suicide

*“I was a bit reluctant to talk about suicide to patients. I think training challenged the myth that raising this issue with someone would cause them to start thinking about it when they weren’t thinking about it before.”*

The ASIST programme’s emphasis on open discussion and a person-centred approach appeared to give these participants permission to speak about suicide, lessening their fears. Many participants said this ability to talk openly was the most beneficial element of the training and the key to preventing suicide. According to Sylvia,

*“Talking to people and being able to have that kind of open conversation with someone about suicide [is the key to preventing suicide] (...) it’s the thing of it takes the air out of the room, but it also like fills it with something else that’s like, ‘Oh, okay, you’re actually giving a shit about this’”.*

These findings suggest the training could help challenge misconceptions about suicide and increased participants’ confidence in initiating open conversations with service users.

#### Value of discussion: Learning from NHS-specific examples.

Participants frequently highlighted the value of experiential learning during the training, which allowed them to learn from one another through NHS-specific examples. In contrast, the taught elements of the training were, at times, seen as *“basic”* and *“repetitive”*. Sam said, *“I don’t think the training gives you that which I think sometimes clinicians want. They want real-life cases and stuff”*.

In recognising the value of learning from one another, several participants suggested the Champions could create their own training. The new training would be the same duration as the more general ASIST programme but with further NHS-specific examples and greater opportunities for group discussions and role-plays. The NHS examples could address high-risk clinical groups and staff wellbeing when working with suicide. James, a Counselling Psychologist, proposed the training should address suicidality in complex cases of trauma as they encountered in their work. Further, Mark suggested that instead of the ASIST’s *“always ask approach”* the Champions should be encouraged to monitor their wellbeing to avoid them beginning suicide prevention interventions they did not have the time or capacity to finish.

While overall feedback around the ASIST training was positive and participants enjoyed sharing applied examples with one another, several participants wished to extend this learning by creating their own NHS-specific training in the future.

### Experience of the role

The domain ‘Experience of the role’ captures how participants navigated the Champion position within their organisational context and their suggestions for future development.

#### Community of support.

Some participants believed that becoming a Champion gave them access to a community of support. During monthly CoP meetings, Champions can offer each other feedback and advice. As Anton said, *“We are talking (...), and you think, ‘Oh yeah, actually I have my ones doing the same thing – but how did you manage?’. This is how we manage, so we share experiences”*.

One participant also mentioned finding support between the CoP meetings through an email thread with all the other Champions. Mark described it as being

*“part of a community that I feel like is talking about this work without shame, with as honest a kind of representation as we can do. So we can support each other. That I feel has been vital because I feel there’s nothing else like it that exists really within the Trust where you can talk about these issues without stigma and without shame.”*

The Champion network and Community of practice appeared to foster peer support and enable shared learning among participants.

#### Organisational issues: Time commitment and scheduling clashes.

Participants generally viewed the two-day time commitment of the ASIST programme positively, appreciating its suitability to the subject matter and the opportunity to get familiar with each other. Yet, several Champions had trouble attending the subsequent CoP meetings due to scheduling clashes.

To improve attendance, the day, time, and format (online or in-person) of CoP meetings alternates each month. Sylvia, a participant in a managerial position, said she was willing to support her staff in attending the meetings. Still, Sam expressed he could not prioritise the meetings over his clinical work, and Chloe felt guilty for not attending as much as she would have liked.

These findings suggest that while participants valued ongoing engagement with the Champion network, workload and scheduling pressures could limit participation. To address time commitment issues, some participants suggested recording meetings. While not a substitute for attendance, the recordings could alleviate Champions’ guilt when missing a session, preserving the mix of online and in-person meetings for accessibility and engagement.

#### Organisational issues: Desire for clarity and structure.

The Champions appeared to particularly value clarity and structure. A few participants found the ASIST’s clear, colour-coded framework helpful for sharing their learning with other members of their team, describing it as *“digestible”* and *“easy to communicate”*.

However, a lack of clarity and structure around the Champion role and its requirements was a source of frustration among interviewees. Several participants struggled to see how the role differed from the work they were already doing. Tina, a HCP, admitted, *“It’s not quite clear what my role is (...) I’ve read a bit, but you know, perhaps that’s this support - I need a bit more understanding of that”*. Some participants attributed this uncertainty to the novelty of the position and believed it would be naturally reconciled as the role developed over time. Other participants felt the uncertainty stemmed from similarities between their clinical work and their work as a Champion and did not negate the value of the latter. As Sam said, *‘Look when I think about the role, I’m not 100% clear on what exactly the role is (...) I think it’s a good idea generally, it’s just an issue of the team I’m working in, it’s such a daily thing. I still think it’s important that it’s rolled out.*

These accounts highlight the need for clearer role definitions to support Champions in understanding, and communicating, how their responsibilities differ from existing clinical duties. Nevertheless, participants felt the skills learned in the training and in the role were transferrable to their day-to-day work.

### Developments

#### Promotion within teams.

Participants described variation in the extent to which they felt their team valued their Champion role. Working in an area where suicidality is common among service users, Holly said she was regularly given time to speak about her role and share updates on suicide prevention guidance in meetings. However, in another area of the Trust also with high levels of suicidality, Tina felt the value of her role was diminished

*“because everyone thinks they deal with it anyway, it doesn’t really, you know… doesn’t really fit.”*

To increase support for the Champion role, several participants suggested the leads could visit different departments to explain the purpose and responsibilities of the role. Sylvia highlighted that this style of promotion could enhance people’s knowledge of the role and its remits across the Trusts, *‘I think it’s about the people knowing about it and knowing, like, what the role is. I think, like in any organisation, it’s about how we promote those roles, but it’s also about people understanding what the roles are. So, it’s not just like, “This person is suicidal. You need to talk to them because you’re the Suicide Prevention Champion.” It’s more about supporting people with those conversations and being there if they don’t feel confident in it, to role model that for them, but it’s like suicide prevention is everyone’s business, so, yes’.* Others recommended auditing team meetings to ensure Champions were given dedicated time to share updates and promote suicide prevention practices within their teams. These findings reflect a desire of certain Champions for greater promotion and awareness of their role across the Trusts to ensure its integration within teams.

#### Dynamic approach.

Given the uniqueness of each suicide case, participants emphasised the need for a dynamic approach to developing the Champion role. Suggestions included continuing to share current research and input from people with lived experience in the CoP meetings and offering retraining opportunities every few years. Mark commented that the Champion role should consistently change and evolve in its engagement with other organisations and the wider community *‘if we are in champion roles and we are being considered champions, then we need to be let out into the world to do the champion work, we need to be collaborating with other organisations that are involved in these interventions that just not involved during the training or supported while they’re having it, because at the end of the day, as we said, if we’re not all singing from the same hymn sheet, then this message gets lost along the way’.*

The novelty of the role was also emphasised as a reason to monitor the role and its support during the initial years of implementation and to make corresponding changes flexibly. As Anton said

*“I think this setup now is something new that started. Probably as we go along, we might be able to pick up whether we need a bit more or we need less in this area.”*

## Discussion

### Main findings

In this study we explored NHS staff experiences of the voluntary “Suicide Prevention Champion” role initiated within two London mental health Trusts. The findings indicate that while the role is generally perceived as meaningful, its implementation was affected by professional barriers and organisational constraints. The following discussion evaluates these findings in the context of the wider suicide prevention literature with a view towards possible future directions and practical implications.

A central finding was that passion—often related to lived experiences of suicide - was a key driver of engagement. Many participants described both personal and professional exposure to suicide as motivating their involvement, echoing literature that claims suicide is a prevalent issue in the NHS and British society [3]. The proportion of first-hand experiences of suicide may have been higher in our sample than the general population due to research that many people considering suicide visit healthcare services before death and certain healthcare staff are at a higher likelihood of expressing suicidal ideation themselves [3,21]. Alternatively, the proportion may be higher due to self-selection bias, as both participation in our sample and the Champion role was voluntary. It is not possible to evaluate why such a large proportion of our sample had encountered suicide from our data or whether it was beyond that which would be expected in the general population. Nevertheless, the presence of a group of staff members passionate about suicide prevention is promising for the viability of the Champion position.

These strong themes of passion and personal experience identified in our study highlight the importance of integrating structured support mechanisms into suicide prevention initiatives. As Croft et al. [20] noted, patient suicides can have a detrimental impact on the wellbeing of NHS staff and some of our participants reflected on their personal experiences of suicide, recognising that talking about suicide would never be easy. The CoP was noted as an important source of support by participants. In contrast to other coping skills, like talking to family, the CoP enabled the Champions to speak to others who understood their work while maintaining confidentiality. The CoP also offered support across the spectrum of suicidality, unlike psychological support for NHS employees, which is often provided only once a death by suicide has occurred.

Several participants, however, found it challenging to attend CoP meetings due to scheduling clashes. This difficulty appeared to be related to workloads as one Champion explained they could not prioritise the meetings over their clinical work. Awenat et al.’s study of inpatient staff suggested that managing the high workloads typical in healthcare may be particularly difficult following a patient’s suicide [18]. As such, Champions who need the support of the CoP may struggle to access it. Strategies in place to mitigate this included alternating dates, times and modes of delivery of meetings to enable more Champions to access the CoP and an email chain which Champions could use to contact one another and share resources as a supplement to the meetings. Although, as the email chain was only mentioned once, it is unclear how widely implemented it is.

In terms of training, the participants generally reported increased confidence and self-efficacy following the ASIST programme. This finding is compatible with the existing research and extends the findings of Magness et al. in America and Campbell and Callary in Glasgow to a London-based sample [24,25]. The ASIST programme appeared to improve staff confidence in two ways. Firstly, in line with previous research, some healthcare professionals in our study were afraid to discuss suicide with service users before they engaged in the suicide prevention training [17,18]. However, the ASIST programme extended beyond conducting risk assessments to focus on the importance of open discussions about suicide. This emphasis on communication seemed to give participants permission to talk about suicide, increasing their confidence. Secondly, attending the training reassured staff that they were up-to-date on the latest suicide prevention guidance. This reassurance seemed particularly important to staff who had completed professional training a long time ago and those who had lost patients to suicide.

Interactive components of the training, including role-plays, which allowed participants to learn from each other, and the experiential elements, including the group discussions, where they could hear about applied NHS-specific examples were also specifically praised by members of our sample. The ASIST’s colour-coded suicide prevention framework was also acknowledged by several participants as an easy and digestible way to share their learning with their wider team. However, some interviewees felt certain didactic elements of the ASIST programme were “basic” and “repetitive”. This finding is understandable given that the ASIST programme was developed for the general population while our sample consisted solely of healthcare professionals. Considering these findings together may indicate that standardised programmes such as ASIST could be effective in improving general competencies, such as staff confidence, and sharing learning with wider teams. However, some contextual adaptation may be valuable in healthcare settings, such as the NHS, to include specific clinical examples.

Interestingly, the study also identified a theme of organisational and professional barriers that limited access to the Champion role and training. Inconsistencies in eligibility criteria and reliance on managerial approval disproportionately affected non-clinical and junior staff. These professional barriers appear to contrast with UK suicide prevention guidance which emphasises the importance of broad “gatekeeper” training across professional groups and equipping a wide workforce to identify and respond to risk [3,12]. The present findings therefore point to a potential implementation gap: while policy frameworks advocate inclusivity, organisational practices may inadvertently restrict access, thereby limiting the reach and equity of suicide prevention efforts. The implementation of the role and how it can be extended is further discussed below.

### Strengths and limitations

A major strength of this study is its applicability. The study was conducted within a year of the Champion role’s establishment, allowing possible issues to be noted and feedback to be incorporated before the role is potentially extended to more teams and Trusts. Enabling the Champions to co-produce the role’s focus in this manner was seen as important for the role’s sustainability by the NHS. Yet, conducting the study so early in the role’s development limited our recruitment efforts as only 60 participants had enrolled as Champions at the time interviews commenced and our sample is small with 10 participants and lacked diversity as most participants were British females. Participants were also at different stages of the role’s commencement at this point. Including all of their experiences offered an insight into different stages of the training and the role. However, as one participant had not begun the ASIST programme and another had yet to attend any CoP meetings, they could not answer related questions, limiting the amount of data that could be gathered.

### Implications for future research

Future research could extend the focus of this study by assessing service user experience. Specifically, a comparative study could examine the experiences of service users seeking support for suicidality from a team with a Champion to those in Trusts where the role was yet to be established, enabling some conclusions about the potential effectiveness of the role to be drawn. Additional qualitative data from the Champions’ coworkers would also be valuable in exploring the acceptability of the intervention.

Another interesting avenue for future research would be to replicate this study in Trusts where the Champion role and training are more developed. This replication would offer an insight into the Champion’s experiences when they are more established in their role and could also monitor whether the developments noted by participants in this study were implemented.

### Developments for the role and training

Assessing the Champions’ experiences of the development and delivery of the role and training indicated five primary avenues for improvement.

1. *Where financially feasible, extending access to the ASIST programme may benefit staff outside of the Champion role.* Participants generally felt the ASIST programme was a useful introduction to suicide prevention, which all staff could benefit from. Where financially feasible, allowing staff to access the ASIST programme without requiring them to become a Champions could improve suicide prevention knowledge across the NHS and could be particularly valuable in areas with high levels of suicidality.
2. *Modify the ASIST programme with input from current Champions for the purpose of the Champion role.* In tandem with this, some participants suggested the ASIST programme could be adjusted for the Champion role to tailor its relevance and applicability to the NHS. The adjustments could be informed by existing Champions in recognition of their varied training needs and the diverse services and risk profiles they serve. In practice, the new training could specifically address applied NHS examples, including high-risk clinical groups and staff wellbeing when working with suicidality. Further opportunities for group discussions and role-plays could also enable the Champions to learn from each other.
3. *Continue to alternate modes of CoP meetings from online to in-person and record meetings for those who cannot attend.* Participants generally appreciated the combination of in-person and online meetings. Online was seen as more accessible while in-person was considered more engaging. Recording the meetings was also suggested to allow Champions to catch-up when they could not attend.
4. *Regularly review and share a description of the role and its responsibilities with the Champions.* The leads of the CoP in these Trusts had created a written statement clarifying the purpose and responsibilities of the Champion role. As the role is constantly developing, such information should be regularly reviewed and shared in CoP meetings.
5. *Promote and Audit the Champion role.*To increase support for the role across the NHS, participants suggested that CoP leads could promote the role, monitor uptake across different departments and audit whether it was being given time in team meetings.

## Conclusion

The range of experiences expressed in this study demonstrates the complexities of suicide prevention training and the importance of considering the experiences of the recipients of that training. The Champion role and ASIST programme demonstrate promise and were generally well-received by participants. Allowing staff to co-produce the focus of the role by incorporating the developments outlined within this study and adopting a dynamic approach to suicide prevention is hoped to ensure the viability of this novel role as it is extended to other NHS Trusts.

## Supporting information

S1 File Interview guide. (DOCX)
