# Supplementary material for: The experiences of ‘Suicide Prevention Champions’ in the UK National Health Service (NHS): A qualitative investigation
Source: PLOS Ment Health. 2026 Aug 10;3(8):e0000623. doi: 10.1371/journal.pmen.0000623 (PMC13456274; doi:10.1371/journal.pmen.0000623)
Supplement: Supplementary file 1 [file pmen.0000623.s001.docx]

Interview Guide

All questions are optional. Participants will be made aware they do not have to answer every question and can withdraw at any time.

**Sociodemographic questions (5 minutes)**

1.	What is your gender?

2.	What is your age?

3.	What is your ethnicity?

Asian or Asian British

- (Indian, Pakistani, Bangladeshi, Chinese, Any other Asian background)

Black, Black British, Caribbean or African

- (Caribbean, African, Any other Black, Black British, or Caribbean background)

Mixed or multiple ethnic groups

- (White and Black Caribbean, White and Black African, White and Asian, Any other Mixed or multiple ethnic background

White

- (English, Welsh, Scottish, Northern Irish or British, Irish, Gypsy or Irish Traveller, Roma, Any other White background)

Other ethnic group

- (Arab, Any other ethnic group)

4.	What is your job title? ________

5.	How many years of clinical experience would you say you have? _________

**Questions (40 minutes)**

*Before the role/training*

Where did you first hear about the ‘Suicide Prevention Champion’ role?

What drew you to the ‘Suicide Prevention Champion’ role?

Before beginning this role, had you experienced suicide in your professional work?

Consider what you thought the ‘Suicide Prevention Champion’ role would be like when you first heard about it. Do you think the role is what you expected?

What obstacles did you anticipate in taking on this new role?

What was your experience of the application process?

*Training*

How did you find the time commitment of training for the ‘Suicide Prevention Champion’ role?

What about the training did you find helpful?

What about the training did you not find helpful?

What aspects of the training did you find challenging?

- How did you cope with that?

How ready did you feel to take on the ‘Suicide Prevention Champion’ role after the training?

What other training do you feel you would benefit from?

Are there any other supports you believe you would benefit from?

*Role*

How has the role of ‘Suicide Prevention Champion’ influenced your workload?

How has the role of ‘Suicide Prevention Champion’ influenced your personal life?

How has the role of ‘Suicide Prevention Champion’ influenced your working relationships with your team?

How has the role of ‘Suicide Prevention Champion’ influenced your working relationships with members of the wider organisation?

How has the role of ‘Suicide Prevention Champion’ influenced your interactions with service users?

What challenges have you experienced as a ‘Suicide Prevention Champion’?

What has supported you as a ‘Suicide Prevention Champion’?

Overall, how would you say your experience as a ‘Suicide Prevention Champion’ has been?

What changes you would like seen made to the ‘Suicide Prevention Champion’ role?

How would you like to see the ‘Suicide Prevention Champion’ role develop?
