# Supplementary material for: The experiences of ‘Suicide Prevention Champions’ in the UK National Health Service (NHS): A qualitative investigation
Source: PLOS Ment Health. 2026 Aug 10;3(8):e0000623. doi: 10.1371/journal.pmen.0000623 (PMC13456274; doi:10.1371/journal.pmen.0000623)
Supplement: Supplementary file 2 [file pmen.0000623.s003.docx]

Professor Jo Billings

UCL Division of Psychiatry

Wing A, 4^th^ Floor, Maple House

149 Tottenham Court Road

London W1T 7NF

Dr Robert C Dempsey

Academic Editor, PLOS ONE

28^th^ April, 2026

Dear Dr Dempsey

**The experiences of ‘Suicide Prevention Champions’ in the UK National Health Service (NHS): A qualitative investigation**

Thank you to you and your reviewers for your detailed and helpful feedback on our manuscript. We have provided a point-by-point response to how we have addressed each comment in the table below.

Thank you for considering our paper. We hope these amendments address the points raised and look forward to hearing further from you.

With kind regards

**Miss Abbie McDonogh and Professor Jo Billings**

| **Comments** | **Author’s Response** | **Manuscript page number** |
| --- | --- | --- |
| *Journal requirements* | | |
| 1. Please amend your detailed Financial Disclosure statement. This is published with the article. It must therefore be completed in full sentences and contain the exact wording you wish to be published.  i. Please clarify all sources of financial support for your study. List the grants, grant numbers, and organizations that funded your study, including funding received from your institution. Please note that suppliers of material support, including research materials, should be recognized in the Acknowledgements section rather than in the Financial Disclosure.  ii. State the initials, alongside each funding source, of each author to receive each grant. For example: "This work was supported by the National Institutes of Health (####### to AM; ###### to CJ) and the National Science Foundation (###### to AM)." iii. State what role the funders took in the study. If the funders had no role in your study, please state: “The funders had no role in study design, data collection and analysis, decision to publish, or preparation of the manuscript.” iv. If any authors received a salary from any of your funders, please state which authors and which funders. | This statement has been amended. | 31 |
| 2. We notice that your supplementary figures are uploaded with the file type 'Figure'. Please amend the file type to 'Supporting Information'. Please ensure that each Supporting Information file has a legend listed in the manuscript after the references list. | Supporting Information has been labelled appropriately and added to the manuscript. | 37 |
| 3. In the online submission form, you indicated that “Pseudoanonymised data is managed by Professor Jo Billings to protect against the risk of identification our participants may face in their workplace. To access the pseudoanonymised data, please email Professor Billings at ([j.billings@ucl.ac.uk](mailto:j.billings@ucl.ac.uk)).”  All PLOS journals now require all data underlying the findings described in their manuscript to be freely available to other researchers, either  1. In a public repository,  2. Within the manuscript itself, or  3. Uploaded as supplementary information.  This policy applies to all data except where public deposition would breach compliance with the protocol approved by your research ethics board. If your data cannot be made publicly available for ethical or legal reasons (e.g., public availability would compromise patient privacy), please explain your reasons by return email and your exemption request will be escalated to the editor for approval. Your exemption request will be handled independently and will not hold up the peer review process, but will need to be resolved should your manuscript be accepted for publication. One of the Editorial team will then be in touch if there are any issues.  If the reviewer comments include a recommendation to cite specific previously published works, please review and evaluate these publications to determine whether they are relevant and should be cited. There is no requirement to cite these works unless the editor has indicated otherwise. | We are not able to share the original data from this study (interview transcripts) as this information is personal and would contravene our ethical approval. We have updated the online submission and communicated this information by email. |  |
| *Editor’s comments* | | |
| 1. Introduction / Suicide Prevention statistics - please could you update the suicide prevalence and prevention statistics as outlined at the start of the introduction (some sources are over 10 years old or statistics from over 5 years ago are referred to here). | These have been updated. | 2-7 |
| 2. Suicide risk - there is a brief discussion of how clinical staff can determine risk in the Introduction, however there could be a better acknowledgement of the issues and challenges associated with suicide risk assessments (e.g., given the NICE guidance on 'do not do', and various UK studies demonstrating that suicide risk is being assessed in unreliable and inappropriate ways). | A new section has been added | 4 |
| 3. In relation to this, it would be useful to have some acknowledgement/brief discussion of when and where in the UK health system suicide risk assessments are likely to be conducted - noting that these are likely to be conducted in various settings. For example, such assessments might be conducted in primary care (e.g., GP appointments) through to more specialist NHS mental health trusts. A better acknowledgment of the 'when' and 'where' such assessments are likely to occur would be useful, as would a bit more clarity in the Introduction re: the structure and nature of the UK NHS as a healthcare system (considering the international nature of our readership). | A new section has been added. | 4 |
| 4. The procedure of the interviews (Pages 6-7) read a little oddly due to the way in which the sentences and paragraphs were split - please pay attention to this in a revision. | This has been rephrased. | 8 |
| 5. Please also provide some brief summary details of the duration of the interviews (e.g., mean/range minutes). | This information has been moved to a place where it is more clear. | 13 |
| 6. I would suggest including a table of the interview questions in the main body / Methods section, rather than the supplementary materials. | Thank you for your suggestion, we attempted to move the table into the methods section but given its length, we decided to keep it in the supplementary materials. This is consistent with what is usually requested by PLOS ONE. | N/A |
| 7. A minor point, but please ensure that units of measurement for age (i.e., years) is added in the respective parts of the manuscript. | This information has been added. | 12 |
| 8. It would be nice to have a table of the participants' demographics, if possible without breaching confidentiality - i.e. each participant listed on their own row, with details (as appropriate) of their role, level of experience. A summary of demographic characteristics (age/ethnicity) can be saved for a written summary in the main text. It would also be useful to have a clearer statement for each participant in terms of their level of experience in the Champion role. | We agree that a table describing participants’ experience and demographics would be a nice addition to the paper, however, to describe participants’ individual demographics alongside their job title or level of experience was considered a significant risk to anonymity. Similarly, as the study is centred in two NHS mental health trusts, describing participants’ demographics was also considered a risk to anonymity. We have managed this risk by presenting aggregated demographic data and using pseudonyms (discussed further below). | N/A |
| 9. Some further details on the reflexive TA approach would be welcome, including further citations to key work by Braun and Clarke - for example, in the Methods, it could be clearer whether this was more broadly an inductive, data-driven rTA or more deductive by nature. | More information on the inductive/deductive themes have been included. | 13 |
| 10. I would appreciate a more developed reflexivity given the nature of the data and the characteristics of the authors/analysts. This does not need to be further details of personal experiences of bereavement by suicide, but a clearer sense of the positionality of the researchers, how any pre-conceptions were considered, whether there were any prior relationships existing between interviewers and participants (e.g., as colleagues), as well as any broader ethics-related considerations. The reflexivity as present is useful but brief. | The reflexivity section has been further developed. | 10-11 |
| 11. Table 2 - I found the number and nature of the deductive/inductive themes to be a little confusing to follow, especially given the relative lack of detail earlier in the manuscript regarding the analysis approach. The 'domain' level in the table was also unclear to me given that rTA usually functions around superordinate themes and subthemes - please consider revising how the domains/themes are presented here as this is a little confusing to follow. | We have revisited the themes and discussed this extensively as a team but did not feel it was possible to re-organise them into another manner without losing the integrity of the original analysis. ‘Superordinate’ themes are usually associated with IP, not reflexive TA (Braun & Clarke, 2020). We have attempted to be clearer in re-stating that the domains were created deductively for the organisational purpose of guiding our readers through the analysis. We hope that will be much clearer. | N/A |
| 12. I would appreciate a clearer overview in the main text about the themes (i.e., a summary of what these overall themes are, and how these themes inter-relate), before progressing to each theme in turn.  In relation to this, a brief summary paragraph/conclusion to each theme would also be helpful - in places, sub/themes are presented rather abruptly. | A larger overview and smaller summaries/conclusions have been included. | 13-23 |
| 14. When presenting each domain/theme in turn, please ensure that a brief summary/description of the theme is presented (e.g., this article may give a better indication of what I am suggesting for the presentation of themes: https://journals.plos.org/mentalhealth/article?id=10.1371/journal.pmen.0000258) | A description has been included in the larger overview. | 13-23 |
| 15. I don't have any specific comments for the Discussion, except this section feels a little rushed and could better cite a range of relevant literatures given the topic/sample. The manuscript as a whole does seem to have missed a range of UK specific literature on suicide prevention in MH trusts and recent developments - this is something one of the reviewers, rightly, critiqued the manuscript in terms of literature coverage. | The discussion section has been reviewed with additional literature and more detailed discussion added. | 24-29 |
| *Comments from Reviewer #1* | | |
| 16. Overall- this service evaluation is not providing significant new knowledge. The rationale for the work: The background rationale for the work is outdated and relies on suicide prevention data, policy and strategy that is not current. It takes a rather uncritical risk focused approach contrary to current preferred practice. The evidence review seems to have missed a large body of existing published research focused on the efficacy of training in suicide prevention. This may be due to the author's focus on risk rather than on the more current 'safety planning' approaches. To be publishable, the background would require significant update including an update on the suicide data presented and the systematic review by Zalsman et al 2016 which contains data from 2006-2016- the author's should check if anything more recent is available. I wonder if a more rigorous literature review is needed? | In light of this feedback we have taken the opportunity to significantly update the introduction to the paper. | 2-7 |
| 17. The intervention seems to be 'Research Champion' yet this is not fully outlined. There is a significant focus on the ASIST training- yet this is also not fully outlined. The CoP is described but it is not fully clear what the link is between the Champions role and CoP membership. Authors should describe the Champion role and the ASIST training more precisely- perhaps using the TIDiER framework? | The section describing the Champion role and ASIST training has been rewritten according to the TIDiER framework. | 6-7 |
| The methods are mostly described although there are some punctuation and presentation errors. Clearer description of the aim and objectives and how the data met these would be helpful. The topic guide is not fully clear. The CoP working on the topic guide seems unclear- were the CoP members also participants? The use of deductive analysis is not outlined in the methods. | Punctuation and presentation errors have been amended. The CoP lead’s role in developing the topic guide and use of deductive analysis has also been clarified. | 8-9 |
| 18. Ethical approval and evaluation registration are reported. Given the small sample, it needs to be made much clearer if the data are presented using pseudonyms. Given that some participants were the only one from specific job roles- perhaps these should not be used as identifiers- perhaps risks confidentiality breach? | The use of pseudonyms has been made clearer. In consulting with the team, referring to participants by their job roles was deemed to be important for reader clarity and was not considered to be a risk to confidentiality given that participation in our study was private and many others were trained in each of the roles across the two Trusts. | 8 |
| **1**9. The findings are reported according to the stated Domains, themes and sub-themes. Some sub-themes are rather thin and lack depth or breadth. | Quotes and detail have been added to thicken the themes. | 13-23 |
| 20. Conclusions: Similarly, I'm not convinced that the conclusions add to the current body of knowledge. With a more precise and focused rationale at the beginning of the paper- and perhaps a more critical and up-to-date stance on the evidence supporting suicide prevention interventions- this work would have more impact. | A more critical and up-to-date stance on the suicide prevention intervention literature has been added to enhance the conclusions and impact of the work. | 30 |
| 21. Recommendations: Should include future implementation methods to monitor uptake and effect of the champion role in a more rigorous way. | This has been added. | 28-29 |
| *Comments from Reviewer #2* | | |
| 22. It's a novel concept of suicide prevention champions. I only have concerns about the very small sample size. It also lacked diversity as most of the participants were British females. The interviews were semi-structured as well. There are some typos to correct in the final version. | Thank you for your kind feedback. We have noted the small sample size as a limitation. Typos have been amended. | 27 |
