# Supplementary material for: The experiences of ‘Suicide Prevention Champions’ in the UK National Health Service (NHS): A qualitative investigation
Source: PLOS Ment Health. 2026 Aug 10;3(8):e0000623. doi: 10.1371/journal.pmen.0000623 (PMC13456274; doi:10.1371/journal.pmen.0000623)
Supplement: Supplementary file 3 [file pmen.0000623.s004.docx]

Professor Jo Billings

UCL Division of Psychiatry

Wing A, 4^th^ Floor, Maple House

149 Tottenham Court Road

London W1T 7NF

Dr Robert C Dempsey

Academic Editor, PLOS ONE

3^rd^ July 2026

Dear Dr Dempsey

**The experiences of ‘Suicide Prevention Champions’ in the UK National Health Service (NHS): A qqualitative investigation**

Thank you kindly and thank you to your reviewers for all of your detailed and helpful feedback on our manuscript. We have provided a point-by-point response to how we have addressed each comment in the table below.

Thank you for considering our paper. We hope these amendments address the points raised and look forward to hearing further from you.

With kind regards

**Miss Abbie McDonogh and Professor Jo Billings**

| **Comments** | **Author’s Response** | **Manuscript page number** |
| --- | --- | --- |
| *Journal requirements* | | |
| 1. Please amend your online Financial Disclosure statement. If you did not receive any funding for this study, please simply state: "The authors received no specific funding for this work." | This statement has been amended. | Online |
| 2. We have noticed that you have uploaded Supporting Information files, but you have not included a list of legends. Please add a full list of legends for your Supporting Information files before or after the references list. | Supporting Information files have been labelled appropriately and added to the manuscript. | 37 |
| 3. If the reviewer comments include a recommendation to cite specific previously published works, please review and evaluate these publications to determine whether they are relevant and should be cited. There is no requirement to cite these works unless the editor has indicated otherwise. | N/A |  |
| 4. Please review your reference list to ensure that it is complete and correct. If you have cited papers that have been retracted, please include the rationale for doing so in the manuscript text, or remove these references and replace them with relevant current references. Any changes to the reference list should be mentioned in the rebuttal letter that accompanies your revised manuscript. If you need to cite a retracted article, indicate the article’s retracted status in the References list and also include a citation and full reference for the retraction notice. | N/A |  |
| *Editor’s comments* | | |
| 5. I have recommended that you address Reviewer 4's comment re: correcting the duplication of 'times' on Line 512. | This has been updated. | 25 |
| 6. Please do consider the comments raised by Reviewer 3 which highlight some aspects of the manuscript that could be further developed to improve clarity. | These changes are detailed below. |  |
| *Comments from Reviewer #3* | | |
| 7. Abstract and Introduction: Line 51-54 “Interventions that directly address suicide means have a clear role in prevention efforts (9).” Please clarify “means”. I understand the author may be referring to means or methods of suicide, but it is best to clarify for the reader prior to discussing. “Means restriction” also requires defining. | We have clarified this term by adding ‘restricting the methods used for suicide’ alongside the more formal term ‘means restriction’. | 3 |
| 8. Abstract and Introduction: Line 71, citation error. | We believe citation 9 is correct (refers to NICE evidence relating to reducing access to means including in terms of barriers for railway suicides) but have amended the statistic provided to accurately represent reference 10. | 3 |
| 9. Abstract and Introduction: Line 66-75 revise for sentence clarity and flow. | Sentences rephrased to promote clarity and flow | 3 |
| 10. Abstract and Introduction: Good justification as to why experiences of staff is necessary to research. | Thank you for this feedback. | N/A |
| 11. Abstract and Introduction: After reading the introduction it is unclear whether suicide prevention champions are healthcare staff (i.e., nurses) that are trained in suicide prevention, or whether they are volunteers from the general public working in NHS hospitals, or both. For example the results mention they are inpatient mental health professionals working as Suicide Prevention Champions on line 118, while the introduction mentions the role as voluntary and open to the public.. Please briefly clarify the roles, scope of practice, and demographics of suicide prevention champions. Readers would like to quickly identify who they are and what they do. Can anyone receive training to be suicide prevention champions? Do they work directly with patients in hospitals? What is the difference between a psychiatric nurse and a suicide prevention champion? These are some questions that come to mind after reading the abstract and introduction. | We have added a line in the abstract to clarify that this role is taken up by inpatient mental healthcare staff, as well as clarified this in the Introduction section. | Abstract p1 and Introduction p6 |
| 12. Abstract and Introduction: I understand this is a novel role to add in the healthcare system, and is still in the development phase, but consider highlighting existing literature across UK, canada, and US about where they work, what they do, and who can be a suicide prevention champion. As a researcher from Canada, we have suicide prevention ASIST program and have yet to integrate people who do this training into the healthcare system. Rather, they are members of the broader community (As the authors have already mentioned), and the purpose of the training is just for members of the public to be aware rather than prevent suicide in hospitals. Given that Suicide Prevention Champions is clearly a novel and innovative role, readers would be interested in learning who they are and what they do in contrast to existing mental health workers in the healthcare system. | Thank you for this suggestion. We had previously detailed the research from Canada but were advised to make the introduction more-UK specific by previous reviewers of this paper! We did describe the use of the ASIST program in America as a broader gatekeeper intervention. However, as you mentioned, the role (to our knowledge) is not in place in other healthcare settings so cross-cultural comparison is not currently possible. Once the role is more established within the UK, it may be more feasible to evaluate the work of suicide prevention champions cross-culturally. | N/A |
| 13. There is a disconnect between the heading “suicide prevention strategies” and the actual content of the section. There is also limited applicability of this section to the results methodology and findings of this study. Focus on ASIST program and the existing online / in person training supports rather than physician and mental health unit areas of help seeking. The suicide prevention champion training evaluated in your study did not seem to address either areas highlighted in this section (targeting physician visits or mental health discharge), either in the results or discussion sections. These are excellent statistics but they just do not fit into this section. Consider moving them to a different section or the discussion. | Thank you for your comment. This information was added into these sections at the request of previous reviewers of this paper, hence we have not made further edits here. | N/A |
| 14. Overall the introduction is written implying that the study will be evaluating Suicide Prevention Champions experiences in both preventing suicide AND experience from the training in suicide prevention. Please revise for clarity throughout. | We believe this comment refers to phrasings such as “training and delivery” which appear throughout and emerges from a belief that the suicide prevention champion role is solely a training program. We feel it is important to highlight that beyond the ASIST training, the Champions also took part in the Community of Practice and that the role itself constitutes a gatekeeper intervention, as defined in the literature and described in our paper. As participants were recruited at a variety of stages from the role and training, we feel it is appropriate to say that the research is targeted towards understanding their experiences of delivering this novel intervention and of the training itself. | N/A |
| 15. Methodology: Excellent, no further comments. | Thank you | N/A |
| 16. Results: Now in the results it is clear these are healthcare workers, not volunteers from the public. Please ensure consistency in the previous introduction sections of the paragraph, and highlight this briefly in the abstract. | See response to previous points, above. |  |
| 17. Results: Consider adding exploring non-clinical staff as suicide prevention champions in the Implications for Future Research. Since the authors previously mentioned that their data is limited in explaining why the peer support worker had trouble accessing the training, consider future research exploring non-clinical healthcare staff as essential in suicide prevention in mental health units. It could be that patients would prefer to receive support from peer support workers or individuals with lived experience rather than white-coat, clinical staff. | We believe this is a great and valuable suggestion but one that has been addressed in the 1^st^ recommendation in “developments for the role and training” as it is more related to the implementation of the role than research. | N/A |
| 18. Discussion: Line 347 citation style mixup | Line 347 is the results section and there does not appear to be a citation error that we can identify. Perhaps this just appears in the proof. | N/A |
| 19. Discussion: Excellent discussion and connection to existing literature. I appreciate the mention of the difficulty the peer support worker had in accessing the training. I also appreciate mention of the limitations of the data in exploring reasons behind this. | Thank you | N/A |
| 20. Conclusion: “Complexities of working with suicide” seems out of scope for this paper. The participants were not being evaluated for working with suicide, rather evaluated on their experiences with the training. Consider reframing to “complexities of suicide prevention training”. | Amended – thank you. | 29 |
| 21. Conclusion: “experiences of those delivering suicide prevention interventions.” What were the suicide prevention interventions? In the general literature this involves deescalation, built environment alterations, and empathic and compassion-focused clinical care, in addition to early crisis intervention. The authors study evaluated experiences with suicide prevention training. The study was focused on experiences of those going through suicide prevention training, not their experiences delivering suicide prevention training. Delivering suicide prevention training implies the individuals in charge of the training, which is not the case here. | Amended to say recipients. Thank you for your suggestion. | 29 |
| *Comments from Reviewer #4* | | |
| 22. After reviewing the revised version of the manuscript, I consider that the article is of good scientific quality and addresses an important and highly relevant public health topic. The authors have adequately addressed the reviewers’ comments, and the revisions made have significantly improved the manuscript. I would nevertheless suggest one minor revisions: Correct a repetition of the word “times” on line 512 of the manuscript. | Thank you for your kind feedback. We have adjusted this error. | 25 |
