# Supplementary material for: The experiences of ‘Suicide Prevention Champions’ in the UK National Health Service (NHS): A qualitative investigation
Source: PLOS Ment Health. 2026 Aug 10;3(8):e0000623. doi: 10.1371/journal.pmen.0000623 (PMC13456274; doi:10.1371/journal.pmen.0000623)
Supplement: Peer review history

# Peer review: Decision Letter 0

**DOI:** 10.1371/journal.pmen.0000623.r001

**Authors:** Robert Dempsey

8 Feb 2026

PMEN-D-25-00461

The experiences of ‘Suicide Prevention Champions’ in the UK National Health Service: A qualitative investigation

PLOS Mental Health

Dear Dr. Billings,

Thank you for submitting your manuscript to PLOS Mental Health. After careful consideration, we feel that it has merit but does not fully meet PLOS Mental Health’s publication criteria as it currently stands. Therefore, we invite you to submit a revised version of the manuscript that addresses the points raised during the review process.

Please can you address the reviewers' and my comments as detailed below. This includes responding to Reviewer 1, noting that whilst this reviewer gave a 'reject' recommendation, I feel that there is some useful feedback from this reviewer and I would like to highlight that we will obtain new independent peer reviewers upon receipt of a resubmission.

Please submit your revised manuscript by Mar 25 2026 11:59PM. If you will need more time than this to complete your revisions, please reply to this message or contact the journal office at mentalhealth@plos.org. When you're ready to submit your revision, log on to https://www.editorialmanager.com/pmen/ and select the 'Submissions Needing Revision' folder to locate your manuscript file.

Please include the following items when submitting your revised manuscript:

- A letter that responds to each point raised by the editor and reviewer(s). You should upload this letter as a separate file labeled 'Response to Reviewers'.
- A marked-up copy of your manuscript that highlights changes made to the original version. You should upload this as a separate file labeled 'Revised Manuscript with Track Changes'.
- An unmarked version of your revised paper without tracked changes. You should upload this as a separate file labeled 'Manuscript'.

Guidelines for resubmitting your figure files are available below the reviewer comments at the end of this letter.

We look forward to receiving your revised manuscript.

Kind regards,

Robert C. Dempsey

Academic Editor

PLOS Mental Health

Journal Requirements:

1. Please amend your detailed Financial Disclosure statement. This is published with the article. It must therefore be completed in full sentences and contain the exact wording you wish to be published.

i. Please clarify all sources of financial support for your study. List the grants, grant numbers, and organizations that funded your study, including funding received from your institution. Please note that suppliers of material support, including research materials, should be recognized in the Acknowledgements section rather than in the Financial Disclosure.

ii. State the initials, alongside each funding source, of each author to receive each grant. For example: "This work was supported by the National Institutes of Health (####### to AM; ###### to CJ) and the National Science Foundation (###### to AM)."

iii. State what role the funders took in the study. If the funders had no role in your study, please state: “The funders had no role in study design, data collection and analysis, decision to publish, or preparation of the manuscript.”

iv. If any authors received a salary from any of your funders, please state which authors and which funders.

2. We notice that your supplementary figures are uploaded with the file type 'Figure'. Please amend the file type to 'Supporting Information'. Please ensure that each Supporting Information file has a legend listed in the manuscript after the references list.

3. In the online submission form, you indicated that “Pseudoanonymised data is managed by Professor Jo Billings to protect against the risk of identification our participants may face in their workplace. To access the pseudoanonymised data, please email Professor Billings at (j.billings@ucl.ac.uk).”

All PLOS journals now require all data underlying the findings described in their manuscript to be freely available to other researchers, either

1. In a public repository,

2. Within the manuscript itself, or

3. Uploaded as supplementary information.

This policy applies to all data except where public deposition would breach compliance with the protocol approved by your research ethics board. If your data cannot be made publicly available for ethical or legal reasons (e.g., public availability would compromise patient privacy), please explain your reasons by return email and your exemption request will be escalated to the editor for approval. Your exemption request will be handled independently and will not hold up the peer review process, but will need to be resolved should your manuscript be accepted for publication. One of the Editorial team will then be in touch if there are any issues.

If the reviewer comments include a recommendation to cite specific previously published works, please review and evaluate these publications to determine whether they are relevant and should be cited. There is no requirement to cite these works unless the editor has indicated otherwise.

Additional Editor Comments (if provided):

As Editor, based on my review of your submission - I have a number of comments to help improve the submitted manuscript. Please provide a response to the other reviewers - note that we will invite new peer reviewers on receipt of a resubmission.

1. Introduction / Suicide Prevention statistics - please could you update the suicide prevalence and prevention statistics as outlined at the start of the introduction (some sources are over 10 years old or statistics from over 5 years ago are referred to here).

2. Suicide risk - there is a brief discussion of how clinical staff can determine risk in the Introduction, however there could be a better acknowledgement of the issues and challenges associated with suicide risk assessments (e.g., given the NICE guidance on 'do not do', and various UK studies demonstrating that suicide risk is being assessed in unreliable and inappropriate ways).

3. In relation to this, it would be useful to have some acknowledgement/brief discussion of when and where in the UK health system suicide risk assessments are likely to be conducted - noting that these are likely to be conducted in various settings. For example, such assessments might be conducted in primary care (e.g., GP appointments) through to more specialist NHS mental health trusts. A better acknowledgment of the 'when' and 'where' such assessments are likely to occur would be useful, as would a bit more clarity in the Introduction re: the structure and nature of the UK NHS as a healthcare system (considering the international nature of our readership).

4. The procedure of the interviews (Pages 6-7) read a little oddly due to the way in which the sentences and paragraphs were split - please pay attention to this in a revision.

5. Please also provide some brief summary details of the duration of the interviews (e.g., mean/range minutes).

6. I would suggest including a table of the interview questions in the main body / Methods section, rather than the supplementary materials.

7. A minor point, but please ensure that units of measurement for age (i.e., years) is added in the respective parts of the manuscript.

8. It would be nice to have a table of the participants' demographics, if possible without breaching confidentiality - i.e. each participant listed on their own row, with details (as appropriate) of their role, level of experience. A summary of demographic characteristics (age/ethnicity) can be saved for a written summary in the main text. It would also be useful to have a clearer statement for each participant in terms of their level of experience in the Champion role.

9. Some further details on the reflexive TA approach would be welcome, including further citations to key work by Braun and Clarke - for example, in the Methods, it could be clearer whether this was more broadly an inductive, data-driven rTA or more deductive by nature.

10. I would appreciate a more developed reflexivity given the nature of the data and the characteristics of the authors/analysts. This does not need to be further details of personal experiences of bereavement by suicide, but a clearer sense of the positionality of the researchers, how any pre-conceptions were considered, whether there were any prior relationships existing between interviewers and participants (e.g., as colleagues), as well as any broader ethics-related considerations. The reflexivity as present is useful but brief.

11. Table 2 - I found the number and nature of the deductive/inductive themes to be a little confusing to follow, especially given the relative lack of detail earlier in the manuscript regarding the analysis approach. The 'domain' level in the table was also unclear to me given that rTA usually functions around superordinate themes and subthemes - please consider revising how the domains/themes are presented here as this is a little confusing to follow.

12. I would appreciate a clearer overview in the main text about the themes (i.e., a summary of what these overall themes are, and how these themes inter-relate), before progressing to each theme in turn.

In relation to this, a brief summary paragraph/conclusion to each theme would also be helpful - in places, sub/themes are presented rather abruptly.

13. PLOS have a generous word count policy and I would advise that the themes as presented are developed and a clearer narrative is woven around the data. Only one quote is presented in some places for each theme, which makes the analysis appear to be light and lacking in a solid grounding in the original data.

14. When presenting each domain/theme in turn, please ensure that a brief summary/description of the theme is presented (e.g., this article may give a better indication of what I am suggesting for the presentation of themes: https://journals.plos.org/mentalhealth/article?id=10.1371/journal.pmen.0000258)

15. I don't have any specific comments for the Discussion, except this section feels a little rushed and could better cite a range of relevant literatures given the topic/sample. The manuscript as a whole does seem to have missed a range of UK specific literature on suicide prevention in MH trusts and recent developments - this is something one of the reviewers, rightly, critiqued the manuscript in terms of literature coverage.

[Note: HTML markup is below. Please do not edit.]

Reviewers' comments:

Reviewer's Responses to Questions

**Comments to the Author**

1. Does this manuscript meet PLOS Mental Health’s publication criteria? Is the manuscript technically sound, and do the data support the conclusions? The manuscript must describe methodologically and ethically rigorous research with conclusions that are appropriately drawn based on the data presented.

Reviewer #1: No

Reviewer #2: Yes

**********

2. Has the statistical analysis been performed appropriately and rigorously?

Reviewer #1: N/A

Reviewer #2: I don't know

**********

3. Have the authors made all data underlying the findings in their manuscript fully available (please refer to the Data Availability Statement at the start of the manuscript PDF file)?

The PLOS Data policy requires authors to make all data underlying the findings described in their manuscript fully available without restriction, with rare exception. The data should be provided as part of the manuscript or its supporting information, or deposited to a public repository. For example, in addition to summary statistics, the data points behind means, medians and variance measures should be available. If there are restrictions on publicly sharing data—e.g. participant privacy or use of data from a third party—those must be specified.

Reviewer #1: Yes

Reviewer #2: Yes

**********

4. Is the manuscript presented in an intelligible fashion and written in standard English?

PLOS Mental Health does not copyedit accepted manuscripts, so the language in submitted articles must be clear, correct, and unambiguous. Any typographical or grammatical errors should be corrected at revision, so please note any specific errors here.

Reviewer #1: Yes

Reviewer #2: Yes

**********

5. Review Comments to the Author

Please use the space provided to explain your answers to the questions above. You may also include additional comments for the author, including concerns about dual publication, research ethics, or publication ethics. (Please upload your review as an attachment if it exceeds 20,000 characters)

Reviewer #1: Overall- this service evaluation is not providing significant new knowledge.

The rationale for the work:

The background rationale for the work is outdated and relies on suicide prevention data, policy and strategy that is not current. It takes a rather uncritical risk focused approach contrary to current preferred practice. The evidence review seems to have missed a large body of existing published research focused on the efficacy of training in suicide prevention. This may be due to the author's focus on risk rather than on the more current 'safety planning' approaches. To be publishable, the background would require significant update including an update on the suicide data presented and the systematic review by Zalsman et al 2016 which contains data from 2006-2016- the author's should check if anything more recent is available. I wonder if a more rigorous literature review is needed?

The intervention description:

The intervention seems to be 'Research Champion' yet this is not fully outlined. There is a significant focus on the ASIST training- yet this is also not fully outlined. The CoP is described but it is not fully clear what the link is between the Champions role and CoP membership. Authors should describe the Champion role and the ASIST training more precisely- perhaps using the TIDiER framework?

The methods:

The methods are mostly described although there are some punctuation and presentation errors. Clearer description of the aim and objectives and how the data met these would be helpful. The topic guide is not fully clear. The CoP working on the topic guide seems unclear- were the CoP members also participants? The use of deductive analysis is not outlined in the methods.

Ethics:

Ethical approval and evaluation registration are reported. Given the small sample, it needs to be made much clearer if the data are presented using pseudonyms. Given that some participants were the only one from specific job roles- perhaps these should not be used as identifiers- perhaps risks confidentiality breach?

Findings:

The findings are reported according to the stated Domains, themes and sub-themes. Some sub-themes are rather thin and lack depth or breadth.

Conclusions: Similarly, I'm not convinced that the conclusions add to the current body of knowledge. With a more precise and focused rationale at the beginning of the paper- and perhaps a more critical and up-to-date stance on the evidence supporting suicide prevention interventions- this work would have more impact.

Recommendations: Should include future implementation methods to monitor uptake and effect of the champion role in a more rigorous way.

Reviewer #2: It's a novel concept of suicide prevention champions. I only have concerns about the very smal sample size. It also lacked diversity as most of the participants were British females. The interviews were semi-structured as well. There are some typos to correct in the final version.

**********

6. PLOS authors have the option to publish the peer review history of their article (what does this mean?). If published, this will include your full peer review and any attached files.

**Do you want your identity to be public for this peer review?**  If you choose “no”, your identity will remain anonymous but your review may still be made public.

For information about this choice, including consent withdrawal, please see our Privacy Policy.

Reviewer #1: No

Reviewer #2: No

**********

[NOTE: If reviewer comments were submitted as an attachment file, they will be attached to this email and accessible via the submission site. Please log into your account, locate the manuscript record, and check for the action link "View Attachments". If this link does not appear, there are no attachment files.]

-->Figure Resubmissions:

While revising your submission, we strongly recommend that you use PLOS’s NAAS tool (https://ngplosjournals.pagemajik.ai/artanalysis) to test your figure files. NAAS can convert your figure files to the TIFF file type and meet basic requirements (such as print size, resolution), or provide you with a report on issues that do not meet our requirements and that NAAS cannot fix.

After uploading your figures to PLOS’s NAAS tool - https://ngplosjournals.pagemajik.ai/artanalysis, NAAS will process the files provided and display the results in the "Uploaded Files" section of the page as the processing is complete. If the uploaded figures meet our requirements (or NAAS is able to fix the files to meet our requirements), the figure will be marked as "fixed" above. If NAAS is unable to fix the files, a red "failed" label will appear above. When NAAS has confirmed that the figure files meet our requirements, please download the file via the download option, and include these NAAS processed figure files when submitting your revised manuscript.

# Author comment: Author response to Decision Letter 1

**DOI:** 10.1371/journal.pmen.0000623.r002

1 May 2026

Attachment Submitted filename: Response to Reviewers 28.04.26.docx

# Peer review: Decision Letter 1

**DOI:** 10.1371/journal.pmen.0000623.r003

**Authors:** Robert Dempsey

30 Jun 2026

PMEN-D-25-00461R1

The experiences of ‘Suicide Prevention Champions’ in the UK National Health Service: A qualitative investigation

PLOS Mental Health

Dear Dr. Billings,

Thank you for submitting your manuscript to PLOS Mental Health. After careful consideration, we feel that it has merit but does not fully meet PLOS Mental Health’s publication criteria as it currently stands. Therefore, we invite you to submit a revised version of the manuscript that addresses the points raised during the review process.

Please submit your revised manuscript by Jul 30 2026 11:59PM. If you will need more time than this to complete your revisions, please reply to this message or contact the journal office at mentalhealth@plos.org. When you're ready to submit your revision, log on to https://www.editorialmanager.com/pmen/ and select the 'Submissions Needing Revision' folder to locate your manuscript file.

Please include the following items when submitting your revised manuscript:

- A letter that responds to each point raised by the editor and reviewer(s). You should upload this letter as a separate file labeled 'Response to Reviewers'.
- A marked-up copy of your manuscript that highlights changes made to the original version. You should upload this as a separate file labeled 'Revised Manuscript with Track Changes'.
- An unmarked version of your revised paper without tracked changes. You should upload this as a separate file labeled 'Manuscript'.

Guidelines for resubmitting your figure files are available below the reviewer comments at the end of this letter.

As the corresponding author, your ORCID iD is verified in the submission system and will appear in the published article. PLOS supports the use of ORCID, and we encourage all coauthors to register for an ORCID iD and use it as well. Please encourage your coauthors to verify their ORCID iD within the submission system before final acceptance, as unverified ORCID iDs will not appear in the published article. *Only*  the individual author can complete the verification step; PLOS staff *cannot*  verify ORCID iDs on behalf of authors.

We look forward to receiving your revised manuscript.

Kind regards,

Robert C. Dempsey

Academic Editor

PLOS Mental Health

Journal Requirements:

1. Please amend your online Financial Disclosure statement. If you did not receive any funding for this study, please simply state: "The authors received no specific funding for this work."

2. We have noticed that you have uploaded Supporting Information files, but you have not included a list of legends. Please add a full list of legends for your Supporting Information files before or after the references list.

3. If the reviewer comments include a recommendation to cite specific previously published works, please review and evaluate these publications to determine whether they are relevant and should be cited. There is no requirement to cite these works unless the editor has indicated otherwise.

4. Please review your reference list to ensure that it is complete and correct. If you have cited papers that have been retracted, please include the rationale for doing so in the manuscript text, or remove these references and replace them with relevant current references. Any changes to the reference list should be mentioned in the rebuttal letter that accompanies your revised manuscript. If you need to cite a retracted article, indicate the article’s retracted status in the References list and also include a citation and full reference for the retraction notice.

**Additional Editor Comments (if provided):**

Dear Authors,

Thank you for your patience with the review process. Unfortunately, our previous reviewers did not response to requests to re-review, so we needed to obtain further reviewers.

I have recommended that you address Reviewer 4's comment re: correcting the duplication of 'times' on Line 512. This reviewer has made other comments which were not entirely applicable to your manuscript and I have removed these from this response letter.

Please do consider the comments raised by Reviewer 3 which highlight some aspects of the manuscript that could be further developed to improve clarity.

Thank you again for your patience.

[Note: HTML markup is below. Please do not edit.]

Reviewers' comments:

Reviewer's Responses to Questions

**Comments to the Author**

1. If the authors have adequately addressed your comments raised in a previous round of review and you feel that this manuscript is now acceptable for publication, you may indicate that here to bypass the “Comments to the Author” section, enter your conflict of interest statement in the “Confidential to Editor” section, and submit your "Accept" recommendation.

Reviewer #3: (No Response)

Reviewer #4: All comments have been addressed

**********

2. Does this manuscript meet PLOS Mental Health’s publication criteria? Is the manuscript technically sound, and do the data support the conclusions? The manuscript must describe methodologically and ethically rigorous research with conclusions that are appropriately drawn based on the data presented.

Reviewer #3: Yes

Reviewer #4: Yes

**********

3. Has the statistical analysis been performed appropriately and rigorously?

Reviewer #3: N/A

Reviewer #4: N/A

**********

4. Have the authors made all data underlying the findings in their manuscript fully available (please refer to the Data Availability Statement at the start of the manuscript PDF file)?

The PLOS Data policy requires authors to make all data underlying the findings described in their manuscript fully available without restriction, with rare exception. The data should be provided as part of the manuscript or its supporting information, or deposited to a public repository. For example, in addition to summary statistics, the data points behind means, medians and variance measures should be available. If there are restrictions on publicly sharing data—e.g. participant privacy or use of data from a third party—those must be specified.

Reviewer #3: Yes

Reviewer #4: No

**********

5. Is the manuscript presented in an intelligible fashion and written in standard English?

PLOS Mental Health does not copyedit accepted manuscripts, so the language in submitted articles must be clear, correct, and unambiguous. Any typographical or grammatical errors should be corrected at revision, so please note any specific errors here.

Reviewer #3: Yes

Reviewer #4: Yes

**********

6. Review Comments to the Author

Please use the space provided to explain your answers to the questions above. You may also include additional comments for the author, including concerns about dual publication, research ethics, or publication ethics. (Please upload your review as an attachment if it exceeds 20,000 characters)

Reviewer #3: Overview

Dear author, congratulations on contributing to this important and novel area of research. I appreciated learning about the new role of suicide prevention champions and experiences with the training delivered. Below includes feedback, mostly pertaining to clarity and cohesiveness in supporting the research question of interest.

Abstract and Introduction

1.Line 51-54 “Interventions that directly address suicide means have a clear role in prevention efforts (9).” Please clarify “means”. I understand the author may be referring to means or methods of suicide, but it is best to clarify for the reader prior to discussing. “Means restriction” also requires defining.

2.Line 71, citation error.

3.Line 66-75 revise for sentence clarity and flow.

4.Good justification as to why experiences of staff is necessary to research.

5.After reading the introduction it is unclear whether suicide prevention champions are healthcare staff (i.e., nurses) that are trained in suicide prevention, or whether they are volunteers from the general public working in NHS hospitals, or both. For example the results mention they are inpatient mental health professionals working as Suicide

Prevention Champions on line 118, while the introduction mentions the role as voluntary and open to the public.. Please briefly clarify the roles, scope of practice, and demographics of suicide prevention champions. Readers would like to quickly identify who they are and what they do. Can anyone receive training to be suicide prevention champions? Do they work directly with patients in hospitals? What is the difference between a psychiatric nurse and a suicide prevention champion? These are some questions that come to mind after reading the abstract and introduction.

6. I understand this is a novel role to add in the healthcare system, and is still in the development phase, but consider highlighting existing literature across UK, canada, and US about where they work, what they do, and who can be a suicide prevention champion. As a researcher from Canada, we have suicide prevention ASIST program and have yet to integrate people who do this training into the healthcare system. Rather, they are members of the broader community (As the authors have already mentioned), and the purpose of the training is just for members of the public to be aware rather than prevent suicide in hospitals. Given that Suicide Prevention Champions is clearly a novel and innovative role, readers would be interested in learning who they are and what they do in contrast to existing mental health workers in the healthcare system.

7. There is a disconnect between the heading “suicide prevention strategies” and the actual content of the section. There is also limited applicability of this section to the results methodology and findings of this study. Focus on ASIST program and the existing online / in person training supports rather than physician and mental health unit areas of help seeking. The suicide prevention champion training evaluated in your study did not seem to address either areas highlighted in this section (targeting physician visits or mental health discharge), either in the results or discussion sections. These are excellent statistics but they just do not fit into this section. Consider moving them to a different section or the discussion.

8. Overall the introduction is written implying that the study will be evaluating Suicide Prevention Champions experiences in both preventing suicide AND experience from the training in suicide prevention. Please revise for clarity throughout.

–

Methodology

Excellent, no further comments.

–

Results

1. Now in the results it is clear these are healthcare workers, not volunteers from the public. Please ensure consistency in the previous introduction sections of the paragraph, and highlight this briefly in the abstract.

2. Consider adding exploring non-clinical staff as suicide prevention champions in the Implications for Future Research. Since the authors previously mentioned that their data is limited in explaining why the peer support worker had trouble accessing the training, consider future research exploring non-clinical healthcare staff as essential in suicide prevention in mental health units. It could be that patients would prefer to receive support from peer support workers or individuals with lived experience rather than white-coat, clinical staff.

–

Discussion

1. Line 347 citation style mixup

2. Excellent discussion and connection to existing literature. I appreciate the mention of the difficulty the peer support worker had in accessing the training. I also appreciate mention of the limitations of the data in exploring reasons behind this.

–

Conclusion

1. “Complexities of working with suicide” seems out of scope for this paper. The participants were not being evaluated for working with suicide, rather evaluated on their experiences with the training. Consider reframing to “complexities of suicide prevention training”.

2. “experiences of those delivering suicide prevention interventions.” What were the suicide prevention interventions? In the general literature this involves deescalation, built environment alterations, and empathic and compassion-focused clinical care, in addition to early crisis intervention. The authors study evaluated experiences with suicide prevention training. The study was focused on experiences of those going through suicide prevention training, not their experiences delivering suicide prevention training. Delivering suicide prevention training implies the individuals in charge of the training, which is not the case here.

Reviewer #4: After reviewing the revised version of the manuscript, I consider that the article is of good scientific quality and addresses an important and highly relevant public health topic. The authors have adequately addressed the reviewers’ comments, and the revisions made have significantly improved the manuscript.

I would nevertheless suggest one minor revisions:

- Correct a repetition of the word “times” on line 512 of the manuscript.

I would also like to warmly thank and congratulate the authors for their work on this essential topic. Suicide prevention is a major public health concern, and the study of gatekeepers’ knowledge, attitudes, and practices makes a valuable contribution to improving strategies for identifying and supporting individuals at risk.

I particularly appreciated the reflection on clarifying the role of gatekeepers ("Suicide Prevention Champions"), which is a key and often decisive factor in the effectiveness of preventive interventions. This issue is also frequently addressed in training programs for such stakeholders, further highlighting the relevance of this topic.

The focus on examining experiences of suicide prevention interventions is also particularly important, as it helps to better understand implementation conditions, as well as the barriers and facilitators of the effectiveness of existing interventions.

I also look forward to the next steps of the program and the continuation of this important work.

Overall, apart from these minor comments, I consider the manuscript to be well revised and recommend its acceptance.

**********

7. PLOS authors have the option to publish the peer review history of their article (what does this mean?). If published, this will include your full peer review and any attached files.

**Do you want your identity to be public for this peer review?**  If you choose “no”, your identity will remain anonymous but your review may still be made public.

For information about this choice, including consent withdrawal, please see our Privacy Policy.

Reviewer #3: No

Reviewer #4: No

**********

[NOTE: If reviewer comments were submitted as an attachment file, they will be attached to this email and accessible via the submission site. Please log into your account, locate the manuscript record, and check for the action link "View Attachments". If this link does not appear, there are no attachment files.]

-->Figure Resubmissions:

While revising your submission, we strongly recommend that you use PLOS’s NAAS tool (https://ngplosjournals.pagemajik.ai/artanalysis) to test your figure files. NAAS can convert your figure files to the TIFF file type and meet basic requirements (such as print size, resolution), or provide you with a report on issues that do not meet our requirements and that NAAS cannot fix.

After uploading your figures to PLOS’s NAAS tool - https://ngplosjournals.pagemajik.ai/artanalysis, NAAS will process the files provided and display the results in the "Uploaded Files" section of the page as the processing is complete. If the uploaded figures meet our requirements (or NAAS is able to fix the files to meet our requirements), the figure will be marked as "fixed" above. If NAAS is unable to fix the files, a red "failed" label will appear above. When NAAS has confirmed that the figure files meet our requirements, please download the file via the download option, and include these NAAS processed figure files when submitting your revised manuscript.

# Author comment: Author response to Decision Letter 2

**DOI:** 10.1371/journal.pmen.0000623.r004

7 Jul 2026

Attachment Submitted filename: Response to Reviewers 03.07.26.docx

# Editor report: Decision Letter 2

**DOI:** 10.1371/journal.pmen.0000623.r005

**Authors:** Robert Dempsey

9 Jul 2026

The experiences of ‘Suicide Prevention Champions’ in the UK National Health Service: A qualitative investigation

PMEN-D-25-00461R2

Dear Prof Billings,

We are pleased to inform you that your manuscript 'The experiences of ‘Suicide Prevention Champions’ in the UK National Health Service: A qualitative investigation' has been provisionally accepted for publication in PLOS Mental Health.

Thank you for your patience with the peer review process, and I apologise for the delays we encountered.

Before your manuscript can be formally accepted you will need to complete some formatting changes, which you will receive in a follow up email. A member of our team will be in touch with a set of requests.

Please note that your manuscript will not be scheduled for publication until you have made the required changes, so a swift response is appreciated.

IMPORTANT: The editorial review process is now complete. PLOS will only permit corrections to spelling, formatting or significant scientific errors from this point onwards. Requests for major changes, or any which affect the scientific understanding of your work, will cause delays to the publication date of your manuscript.

If your institution or institutions have a press office, please notify them about your upcoming paper to help maximize its impact. If they'll be preparing press materials, please inform our press team as soon as possible -- no later than 48 hours after receiving the formal acceptance. Your manuscript will remain under strict press embargo until 2 pm Eastern Time on the date of publication. For more information, please contact mentalhealth@plos.org.

Thank you again for supporting Open Access publishing; we are looking forward to publishing your work in PLOS Mental Health.

Best regards,

Robert C. Dempsey

Academic Editor

PLOS Mental Health

***********************************************************

Reviewer Comments (if any, and for reference):
